# Evaluating the influence of organizational capability on fundraising success in lebanese non-governmental organizations

**DOI:** 10.1016/j.heliyon.2025.e41891

**Published:** 2025-01-11

**Authors:** Nada Jabbour Al Maalouf, Chadia Sawaya, Jean Elia

**Affiliations:** School of Business, Holy Spirit University of Kaslik (USEK), Jounieh, Lebanon

**Keywords:** *Donor relationship*, *Fundraising*, *Funding conditions*, *Financial management practices*, *Competition*, *NGOs*, *Organizational capability*

## Abstract

**Purpose:**

Given the significant rise in the number of non-governmental organizations (NGOs worldwide, especially in developing nations, this study investigates the factors influencing fundraising levels among NGOs in Lebanon, a region grappling with profound economic and political challenges.

**Methodology:**

Using a quantitative approach, the study analyzes key predictors, including donor funding conditions, financial management practices, organizational fundraising capacity, and competition for funds. Drawing upon a positivist research philosophy and a deductive approach, this study employed a questionnaire distributed via Google Forms. The responses were collected from 124 key decision-makers in NGOs across Lebanon. Statistical analysis using SPSS was employed to investigate the relationships between the variables.

**Findings:**

The findings reveal that competition for funds is a significant determinant of fundraising success, underscoring the intense rivalry among NGOs for limited donor resources. In contrast, financial management practices, organizational capacity, and donor funding conditions exhibit negligible impacts, highlighting a crucial divergence from established literature.

**Contribution and originality:**

This study addresses a critical gap by examining Lebanon's unique context, where external factors significantly influence fundraising dynamics for NGOs. By offering actionable insights for both NGOs and policymakers, the research advocates for tailored strategies that specifically address local challenges. The findings aim to enhance the sustainability and effectiveness of NGOs within Lebanon's complex landscape. Furthermore, this research contributes to the theoretical discourse on NGO fundraising, emphasizing the necessity for context-specific approaches in developing regions.

## 1 Introduction

Non-governmental organizations (NGOs) play a vital role in social change, especially in developing countries where political climates are at stake. They contribute to philanthropic support, poverty eradication, and safety in areas where government capability is often lacking [1,2]. They are usually found in remote or underserved areas, connecting communities and supporting marginalized groups, including refugees, displaced individuals, those with extremely low incomes, and people with disabilities [3]. NGOs can adapt and rapidly respond to public requirements, especially in unstable situations [4].

In the past decade, the number of NGOs has increased by over 200 % due to global economic corrosion in developing countries to reach over 10 million worldwide. In these countries, NGOs need sufficient funding to continue operating and often struggle with a lack of funding due to never-ending demands and limited resources [5]. Thus, competition for funding among NGOs targeting deprived communities has increased [6]. Funding competition is the process where recipient organizations must apply for funds from donors, evaluating applications based on predetermined criteria. Factors such as donor fatigue, operational challenges, and corruption can influence the availability and durability of funding for NGOs. Donors include individuals and entities who can benefit from tax exemptions because of their donations which can be cash donations, in-kind donations, sponsorships and grants, planned giving, and volunteering expenses [7]. The dependency on foreign funding significantly impacts NGOs' activities, leading to fund unpredictability and a lack of sustainable development [8].

Resource mobilization which includes obtaining and managing different resources is crucial for ensuring the sustainability of NGOs [9]. In this aspect, NGOs in Africa and the Middle East face many challenges such as inadequate strategic planning, poor management, inefficient policies and procedures, high staff turnover, and the absence of alternative fundraising methods [3].

Besley and Ghatak [10] highlighted the significance of matching the mission preferences of principals and agents in increasing the organizational efficiency of private nonprofits and public ones. Aldashev and Verdier [11] mentioned that NGOs should assign their time resource to fundraising and working on their project, which draws donations from private sources. They also mentioned that in the case of a mixed-size market, the level of fundraising augments with the number of NGOs. Moreover, the free-entry equilibrium number of NGOs can be more or less than the socially optimal number [11]. This depends on the efficacy of the adopted fundraising technology. If the market size is endogenic and NGOs collaborate in drawing additional funders, the levels of fundraising drop with the NGOs’ number, and the free-entry equilibrium number of NGOs is smaller than the one that increases the well-being of beneficiaries and donors. If NGOs are capable of switching funds for private use, multiple equilibria appear [11].

Furthermore, Aldashev et al. [12] mentioned that there is high fundraising competition among NGOs. Donors have a dormant inclination to give, but they need to be "awakened" to donate. Each NGO decides whether to compete in the same market (clustering) with spillovers or to face weaker competition under issue specialization. The authors found that equilibrium clustering is more probable to happen when the share of multiple-issue donors is moderately large, and when the fundraising technology is adequately efficient. Additionally, this situation is socially inefficient when the cost of fundraising takes intermediate values and the motivation for donors to give is fairly high.

Aldashev et al. [13] mentioned that more transparency on the funds' usage has a vague impact on the total public good provision and funders' welfare. They found that transparency boosts non-profits to curb rent-seeking more energetically inside organizations, but it also slopes the playing field against non-profits facing higher monitoring costs, making them abandon their missions and reducing non-profit multiplicity. In addition, the authors believed that funders' welfare is lower under transparency for intermediate levels of asymmetry in monitoring costs. In the same manner, Kopel and Marini [14] emphasized transparency and mentioned that NGOs have been lately instructed to reveal information regarding the compensation packages of their executives which is available not only to present and potential funders but also to rival NGOs competing for fundraising. The authors found that the observability of incentive contracts decreases existing competition which can be helpful in terms of NGOs' yields and social welfare, specifically when these organizations are stuck in a condition of extreme fundraising actions. Yet, the authors showed that when funders' inclination to donate is sufficiently dissimilar, publicly available contract information can distort the NGOs’ choice of projects, leading to socially inefficient project clustering.

Fundraising levels are impacted by several factors. Bish and Becker [15] emphasized the importance of having a strong organizational capacity to qualify for funding. This includes the ability to plan, administer, operate, and deliver programs and services [15]. Also, donor funding conditions tend to enhance the capacity to secure funding and enhance accountability [16]. Furthermore, financial management practices are necessary to ensure that monetary resources are used efficiently to maintain performance [17]. Those practices include securing loans, seeking investments, executing cost-cutting measures, attracting donations, and engaging in associations.

Based on the significance of fundraising in NGOs, this research aims to examine the effect of organizational capability on the fundraising levels of NGOs in Lebanon; specifically, the effect of organizational capacity, funding conditions, funds competition, and financial management. Lebanon was selected as the focal country for this study due to several reasons. First, Lebanon has witnessed a noteworthy rise in the number of NGOs, driven by ongoing political, economic, and social crises. The number of NGOs and civil society organizations in Lebanon has progressively increased due to the absence of government support in critical sectors. As of June 2024, there are 11,676 registered associations in the country [18]. Second, Lebanon's prolonged economic downturn and political instability have increased the need for NGO services, while simultaneously restricting their access to domestic funding sources. Consequently, Lebanese NGOs are highly dependent on international donors, highlighting the critical role of organizational capability in securing and managing these funds. Moreover, Lebanon's situation mirrors broader challenges faced by NGOs across the Middle East and North Africa (MENA) region, making it a representative case for understanding NGO fundraising dynamics in resource-scarce and crisis-affected environments.

The findings of this research hold significant value for both practitioners and academics. For NGOs, particularly in Lebanon and other developing regions, the insights gained will be instrumental in formulating sustainable fundraising techniques and strategies. Understanding the pivotal role of organizational capability allows NGOs to enhance their internal processes, develop better donor engagement practices, and improve their overall fundraising outcomes. Furthermore, the research provides a valuable framework for NGO leadership and management teams to implement proactive strategies that can sustain the NGO's financial health. This is especially critical given the increasing competition for limited resources in the nonprofit sector.

From a scholarly perspective, this study fills an important gap in the literature. Previous studies such as Amenu [19], Bell and Cornelius [20], Brown and Guo [21], Bryson [22], Crockwell [23], Kyalimpa [24], Letts et al. [25], Light [26], Nwauche and Flanigan [27], Okorley and Nkrumah [28], Peiffer et al. [29], Sobeck and Agius [30], and Tomno [31] have explored various aspects of NGO management and fundraising. However, there remains limited research specifically examining the direct impact of organizational capability on fundraising success within NGOs, particularly in developing countries like Lebanon. Most existing research tends to focus on larger, international NGOs or on external factors such as donor behavior, neglecting the internal organizational competencies that enable effective fundraising. By focusing on Lebanese NGOs, this research addresses the under-explored nexus between internal organizational factors and fundraising capacity in a challenging socio-economic and political environment.

Thus, the findings will contribute new knowledge to the field of nonprofit management by offering a localized perspective on how organizational capability drives fundraising success, and how these insights can be adapted to other similar contexts. This not only helps NGOs in Lebanon but also offers a model that can be applied in other developing nations facing similar constraints. Ultimately, the research provides actionable recommendations for NGOs striving to enhance their fundraising efforts and achieve their long-term objectives.

Based on the objectives, the primary query is as follows: What is the effect of organization capability – organizational capacity, donor funding conditions, competition for funds, and financial management practices – on the level of fundraising by non-governmental organizations in Lebanon?

## 2 Literature review

The literature review included two main sections which are the theoretical background and hypotheses development.

### 2.1 Theoretical background

In the context of NGOs and specifically fundraising, several theories stand as theoretical foundations for this study. These theories include the Agency Theory, Resource Dependence Theory, Stakeholder Theory, Social Exchange Theory, Social Identity Theory, Justice Motivation Theory, and Prosocial Behavior Theory.

#### 2.1.1 Agency Theory

The historical development of the Agency Theory is attributed to Ronald Coase and Michael Jensen in the middle of the 20th century [32]. This theory provides a comprehensive framework to illuminate the intricate dynamics between principals and agents, prevalent across various industries [33]. Mechanisms that align the interests of principals and agents are essential for promoting transparency, accountability, and efficient decision-making [34]. Understanding Agency Theory is imperative for comprehending how individuals and organizations navigate complex decision-making situations. Key concepts in this theory include the roles of principal and agent, information asymmetry, moral hazard, adverse selection, agency costs, and incentive alignment [35]. Understanding and resolving agency issues continue to be vital for attaining organizational goals and safeguarding stakeholders' interests amid increasing complexity and globalization [36,37]. Agency Theory finds applications across diverse fields, such as corporate governance, contractual relationships, nonprofit organizations, and the public sector. In the nonprofit sector, Agency Theory illuminates interactions between donors and organizational leaders, ensuring transparency and accountability [38].

#### 2.1.2 Resource Dependence Theory

Resource Dependence Theory (RDT) is a pivotal framework to be discussed in this study since it sheds light on how organizations navigate their external environments to secure essential resources for sustainability and extension. Jeffrey Pfeffer and Gerald Salancik were the first to discuss RDT back in the 1970s [39]. RDT emerged in response to the limitations of previous organizational theories, providing a paradigm shift in understanding how organizations navigate resource dependencies. RTD elucidates the interdependence between organizations and stakeholders, emphasizing the complexities of resource acquisition and management [40]. RDT asserts that organizations are not autonomous but interdependent entities relying on various external resources, including monetary capital, knowledge, human expertise, technology, and societal legitimacy. Recognizing how resource dependencies can lead to power imbalances, influencing organizational behavior and decision-making, is central to RDT [41]. The basic principles of RDT include resource scarcity, highlighting organizations' constant struggle for essential resources; interdependence, emphasizing the complex relationship between organizations and their external environment; resource dependence, underscoring organizations' reliance on external sources; and resource control, addressing the impact of external control on organizations' autonomy [39,42]. In an era marked by globalization and sustainability, RDT provides valuable tools for addressing the obstacles that organizations face in resource attainment, distribution, and organization [42]. RDT has found applications across various fields, including business, nonprofit sectors, public administration, and global relations. In the nonprofit sector, RDT plays a crucial role in securing funding [43].

#### 2.1.3 Stakeholder Theory

Stakeholder Theory (ST), a well-established framework in organizational studies, advocates that companies must take into consideration the interests of all stakeholders affected by their decisions and obligations to shareholders. ST promotes transparency, ethical decision-making, and responsible behavior [44]. This theory emerged in the 1960s and 1970s and was developed by Edward Freeman and Igor Ansoff. It evolved into a prominent theoretical framework with lasting impacts on corporate behavior, corporate social responsibility programs, and ethical considerations in modern organizations [45]. ST is built on several core principles which are inclusive recognition, diverse stakeholders, relationship management, and balanced prioritization. First, inclusive recognition stresses the importance of organizations acknowledging and considering the interests of all stakeholders impacted by their actions, fostering accountability and moral behavior. Second, diverse stakeholders, representing various individuals and entities, underline the complex nature of stakeholder interactions, necessitating a considerate and comprehensive approach to engagement. Third, relationship management underscores the importance of skillfully managing stakeholder interactions for sustainable success, considering the fluid and multifaceted nature of these relationships. Finally, balanced prioritization motivates companies to uphold equilibrium among the various stakeholder interests [45]. In addition, ST introduces key factors for effective implementation, including stakeholder identification, prioritization, and engagement. Stakeholder identification involves systematically identifying all stakeholders impacting or impacted by an organization's activities, ensuring a comprehensive approach to later engagement strategies. Prioritization involves assessing and ranking the relative significance of stakeholders' interests for more effective resource allocation. Stakeholder engagement is a dynamic process focusing on establishing and preserving positive relationships, contributing to an organization's long-term sustainability, moral behavior, and legitimacy within the intricate web of stakeholder relations [45]. ST has broad applications across various domains. In the context of NGOs, ST can be used to manage complex relationships with beneficiaries, donors, volunteers, employees, and regulatory bodies, resulting in enhanced societal impact and ethical operations [45].

#### 2.1.4 Social Exchange Theory

Social Exchange Theory (SET) is used as a foundation to comprehend fundraising [10]. SET is based on the belief that “voluntary actions of individuals are motivated by the returns they are expected to bring and typically do bring from others,” such as social recognition [46, p. 91]. Relationship marketing is related to SET. It includes “establishing, developing, and maintaining successful relational exchanges” [47, p. 20]. In the context of NGOs, relationship marketing is important to retain good relationships with stakeholders and attract donors [10]. Many scholars such as Cook and Lasher [46], Gächter et al. [47], Hollander [48], and Kelly [49] considered SET when discussing philanthropic responsibility and found that SET increases voluntary cooperation. Thus, the power within the relationships that are built is significant. Fundraising largely includes a social exchange relationship between an NGO and a donor, in which the power of each relative to the other determines the outcome of the exchange [10].

#### 2.1.5 Social Identity Theory

Social Identity Theory (SIT) explains how persons derive part of their identity from the clusters or organizations to which they belong, creating a sense of belonging and loyalty [50]. When people define themselves in relation to an organization, they internalize the organization's values, goals, and identity as part of their own. This connection can foster a strong emotional bond, leading to behaviors that support the organization's success and sustainability [51]. In the setting of alumni engagement, Mael and Ashforth [52] explored how positive institutional identification—where alumni feel a strong connection to their alma mater—significantly influences their likelihood of donating. Alumni who view their former institution as an integral part of their identity are more likely to engage in behaviors that reinforce their connection, such as making financial contributions to support its future growth and success. This concept underscores the importance of fostering a strong emotional bond between alumni and their institutions, as it can be a critical driver of philanthropy and donor retention. Although SIT has not been traditionally used in discussions of fundraising per se, it has been extensively applied in research on philanthropic responsibility. In this domain, persons who identify with a group or cause are more likely to feel a sense of obligation or responsibility to support it, whether through donations, volunteering, or advocacy. This theory helps explain why individuals are motivated to give back to organizations or causes that resonate with their personal or social identity. Therefore, applying SIT to fundraising contexts—especially for NGOs or educational institutions—can offer valuable insights into how cultivating a strong sense of organizational identification can lead to increased donor engagement and sustained financial support.

#### 2.1.6 Personal Donorship Theory

Mount [53] discussed Personal Donorship Theory which posits that donors donate based on five criteria. The first is involvement in the organization. Donors are more likely to contribute financially if they have a personal connection or direct engagement with the organization, such as through volunteering or participation in its activities. This involvement fosters a sense of belonging and investment in the organization's success. The second factor is the alignment of the organization's mission with the donor's personal interests. Donors are more inclined to support causes that resonate with their own values and beliefs. When individuals see a direct connection between their interests and the organization's goals, they are more likely to contribute to further those shared goals. Self-interest is the third criterion, which refers to the personal benefits donors may derive from their contributions. These benefits may include public recognition, enhanced social status, or the personal satisfaction of making a difference. While altruism plays a significant role in philanthropy, self-interest often motivates individuals to continue giving, especially when there are tangible rewards or recognition attached to their donations. Another important factor is disposable income. Donors' financial capacity to give significantly influences their donation behavior. Individuals with more disposable income are more likely to donate larger sums, while those with limited resources may contribute smaller amounts or donate less frequently. Economic conditions and personal financial stability can thus affect an individual's propensity to give. Finally, past donation behavior plays a crucial role in shaping future contributions. Donors who have a history of giving are more likely to continue supporting the same organization, especially if they had a positive experience or witnessed tangible outcomes from their previous donations. This highlights the importance of maintaining strong relationships with existing donors to encourage ongoing support [53]. Thus, Personal Donorship Theory underscores the complex interplay of personal involvement, alignment of values, self-interest, financial capacity, and historical giving patterns in shaping donor behavior. Understanding these factors allows organizations to tailor their fundraising strategies more effectively to meet the motivations of different donor groups.

#### 2.1.7 Justice Motivation Theory

Justice Motivation Theory (JMT) posits that people donate due to injustice motives [54,55]. This theory suggests that people are motivated to correct perceived imbalances or injustices in the world by contributing to causes that address these disparities. The underlying belief is that by donating, individuals can help rectify situations that they deem unfair, thereby restoring a sense of justice in the world. Warren and Walker [56] further expanded on this theory by applying it to philanthropic activities. They found that people who believe in a just world are often motivated to engage in charitable giving because they view their contributions as a way to create fairness or equality. These individuals believe that everyone should receive their due, and when they perceive that some people are disadvantaged or unfairly treated, they feel compelled to act through philanthropy. This behavior aligns with their desire to maintain or restore justice, as donations become a means to balance societal inequalities. JMT highlights that the motivation behind charitable giving is not solely altruistic but is also deeply rooted in individuals' desire to maintain a moral order where fairness prevails. Donating to causes that promote social equity allows individuals to align their actions with their personal sense of justice, giving them a sense of fulfillment and moral satisfaction. In essence, JMT explains that people donate not only out of compassion but also to fulfill a psychological need to live in a just and fair world.

#### 2.1.8 Prosocial Behavior Theory

Fundraising prosocial behavior stems from a psychology-related theory which is the Prosocial Behavior Theory (PBT) which was discussed in many studies such as Clark [57], Dawes et al. [58], Diamond and Kashyap [59], Hogg [60], and Eisenberg and Mussen [61], indicating its robustness and applicability in explaining charitable actions. Prosocial behavior is voluntary actions that aim to help others [61]. The concept of prosocial behavior was introduced by Edward Wilson in 1975 who found that people help each other in many ways by nature since they are humans, not animals [10]. In the context of fundraising for NGOs, PBT is highly relevant as it underpins the motivations behind why individuals choose to support these organizations. By incorporating PBT into this study, we can better explore how altruistic tendencies, driven by the desire to help others, influence donor behavior.

### 2.2 Hypotheses development

#### 2.2.1 Organizational capacity and fundraising levels

Organizational capacity is the ability of an organization to effectively utilize its resources—such as people, processes, systems, and finances—to achieve its goals and mission [62]. It encompasses the organization's competence in areas like leadership, strategic planning, infrastructure, financial management, human resources, and technology. Strong organizational capacity means that an organization is equipped to perform efficiently, adapt to changes, and sustain its operations over time [62]. In the context of NGOs, organizational capacity is crucial for managing programs, attracting and retaining donors, responding to challenges, and achieving fundraising success. It can include the ability to build relationships with stakeholders, develop effective fundraising strategies, and ensure proper governance and accountability [23]. Several studies have examined the concept of organizational capacity within the context of NGOs, emphasizing its crucial role in enhancing both performance and sustainability.

Letts et al. [25] offered a comprehensive framework, underscoring the importance of building a robust infrastructure to support high performance and improve fundraising capabilities. They stressed that without a well-developed organizational structure—including effective leadership, sound financial management, and strategic planning—NGOs may struggle to meet their goals or compete effectively for donor support. Additionally, Light [26] expanded on this idea by exploring how capacity building contributes to the long-term performance of nonprofit organizations. Light's work highlights that consistent investment in organizational capacity can lead to better fundraising outcomes, stronger community impact, and overall organizational sustainability.

Furthermore, Sobeck and Agius [30] conducted an in-depth analysis of the discrepancies between research and practical implementation regarding organizational capacity in nonprofit organizations. To address this issue, Sobeck and Agius [30] proposed a set of strategies aimed at enhancing capacity-building efforts. These strategies include strengthening internal management processes, improving financial management practices, fostering leadership development, and creating more effective governance structures. Additionally, they emphasized the importance of external support, such as partnerships and collaborations with other organizations, to build capacity in resource-limited environments.

Focusing on governance, Brown and Guo [21] conducted an in-depth exploration of organizational capacity by specifically focusing on the role of board effectiveness and governance in nonprofit organizations. Their research highlighted the critical link between strong governance structures and the overall capacity of an organization to function effectively and achieve its mission. They argued that the board of directors plays a pivotal role in shaping an organization's strategic direction, ensuring accountability, and securing resources, all of which are fundamental components of organizational capacity. The authors emphasized that a well-functioning board can significantly enhance an NGO's capacity by providing leadership, oversight, and support in key areas such as financial management, strategic planning, and operational efficiency. The study also underscored that governance practices influence the capacity of NGOs to build relationships with external stakeholders, including donors, partners, and the community.

Also, Okorley and Nkrumah [28] found that proper funding, sound governance, and skilled staff are crucial for NGOs' ability to raise money. Bell and Cornelius [20] mentioned that organizational capacity significantly impacts fundraising efforts. The authors recommended that NGOs invest in grant-fundraising capabilities and diversify their funding sources. Furthermore, Tomno [31] discussed funding for local NGOs and identified factors preventing these organizations from receiving donor funding such as employee retention and governance. Also, Kyalimpa [24] stressed the importance of leadership in funding for NGOs in Uganda.

Crockwell [23] discussed the importance of organizational capacity in community-based organizations and highlighted strategies for capacity building. Amenu [19] examined the opportunities and difficulties faced by NGOs in relation to fundraising practices such as resource competition, community attitudes towards supporting NGOs, organizational capacity, budget constraints, and extreme reliance on foreign donor sources. In the same context, Bryson [22] focused on strategic planning and organizational capacity building in public and nonprofit organizations, providing up-to-date insights into how organizations can strengthen their capacity for better outcomes. Further, Peiffer et al. [29] explored how role ambiguity among board members affects organizational capacity in nonprofits, providing insights into governance and leadership challenges. Also, Nwauche and Flanigan [27] discussed the role of capacity building at different stages of a nonprofit organization's life cycle, offering a contemporary view of how capacity affects long-term sustainability.

Based on the previous studies, the following hypothesis was developed.

H1 Fundraising levels affected by the organizational capacity of NGOs in Lebanon.

#### 2.2.2 Donor funding conditions and fundraising levels

Several studies have explored the relationship between donor conditions and NGO financial stability, with a primary focus on donor dependence and the need for diversified funding sources. Nuka [63] highlighted the instability of NGO finances due to heavy reliance on foreign donors and suggested that NGOs should diversify revenue through membership dues and other sources to ensure sustainability. This aligns with Singh and Singh's [64] emphasis on the importance of trust-building with donors through a strong reputation, selfless service, and addressing pressing societal issues. However, despite this, their study also revealed that many NGOs waste resources on limited groups, leading to increased scrutiny from donors, such as surprise audits and contract termination.

Milelu [3] found that positive relationships with donors can foster accountability, improve communication, and ensure adherence to donor rules, thereby enhancing the financial sustainability of NGOs. This implies that NGO fundraising success may be influenced by how effectively they manage donor relationships and networks. However, the study largely focused on the benefits of established relationships rather than exploring how donor expectations or conditions, such as reporting requirements, affect overall fundraising capacity.

Kabbara and Ozgit [65], in their study on NGOs in Lebanon, illustrated the challenges NGOs face in securing funding, particularly in times of crisis when demands for services increase. Their findings underscored the role of donor conditions in determining the availability of financial resources. They also pointed out that communication among stakeholders and better governmental support are necessary to help NGOs meet donor demands and improve their fundraising capacity.

Despite these insights, existing research still lacks a comprehensive understanding of how specific donor conditions, such as donor preferences for certain projects or the volatility of donor commitment, directly affect fundraising levels. Additionally, few studies provide a quantitative analysis of how varying donor conditions might impact the ability of NGOs to consistently raise funds. Understanding these dynamics could offer practical insights for NGOs to better navigate donor relationships and secure sustainable funding. Therefore, building upon the existing literature, the following hypothesis was proposed.

H2 Fundraising levels of NGOs in Lebanon are affected by donor funding conditions.

#### 2.2.3 Funds competition and fundraising levels

Existing literature highlights the intense competition among NGOs for limited donor funds, particularly in developing regions, where external funding is often the primary source of financial support. Batti [9] examined the challenges faced by local NGOs in Africa, noting that reliance on foreign donors and the stiff competition for these funds often leaves NGOs with insufficient resources to fully implement their projects. This competition hinders their ability to scale up operations, leading to financial instability and limiting the scope of their activities. Similarly, Aden [66] explored the experiences of local NGOs in Somalia, particularly those involved in nutrition projects, and found that funding shortages due to intense competition contributed to corruption, bribery, and a lack of coordination. While NGOs were present throughout the nation, the competition disrupted service delivery and threatened their long-term sustainability. Aden's findings suggest that, while NGOs may be plentiful, the fight for funding can undermine their efficiency and effectiveness, diminishing their independence and long-term viability. Mymunah [67] added to this discussion by arguing that competition for funds among NGOs necessitates the development of robust internal strategies. Mymunah [67] recommended that NGOs focus on creating strategies for fundraising and enhancing their organizational capacity rather than counting on external donors. This recommendation emphasizes the importance of local NGOs building their sustainable revenue models and organizational resilience to mitigate the negative effects of competition.

While these studies have provided valuable insights into the challenges NGOs face in securing funding amidst intense competition, the direct impact of funds competition on fundraising effectiveness remains underexplored. Few studies have investigated the extent to which this competition affects NGOs' ability to raise adequate funds, particularly in contexts where donor fatigue or shifting donor priorities may exacerbate competition. Moreover, while these findings emphasize the need for organizational capacity-building and strategic planning, the literature lacks a quantitative analysis of how the level of competition within the NGO sector directly influences fundraising outcomes. Given these gaps, it is critical to examine how the competition for funds impacts the fundraising capabilities of NGOs, particularly in Lebanon, where the demand for NGO services has escalated due to ongoing crises. Understanding the dynamics of funds competition within the sector will provide insights into how NGOs can adapt their fundraising strategies to remain financially viable. Based on these studies and the gap in the literature, the following hypothesis is proposed.

H3 The fundraising level is impacted by the competition for funding among NGOs in Lebanon.

#### 2.2.4 Financial management and fundraising levels

Effective financial management is critical to the sustainability and success of NGOs, especially in environments where funding sources are limited or volatile. Several studies have underscored the importance of sound financial management practices to ensure that NGOs can maintain stable operations and continue to serve their target populations. Saungweme [68] researched local NGOs in Zimbabwe, finding that the absence of income diversification and proactive fundraising strategies often leads to financial instability, especially when donor funding declines. The study emphasized the need for developing diverse fundraising strategies, income diversification mechanisms, and maintaining strong relationships with donors as key components of long-term financial sustainability. Gyamfi [7] highlighted the significance of sound financial control procedures in local NGOs. According to the research, robust financial controls not only facilitate better decision-making but also ensure that organizational activities align with budget estimates, contributing to the overall sustainability of the organization. Without these controls, NGOs risk mismanaging funds, leading to budgetary shortfalls. Ayene et al. [69] explored the financial management practices of local NGOs and found that these organizations often rely heavily on grants and donations, with limited funds coming from membership fees or other internal sources. The study identified key issues such as the lack of staff participation in budget reviews, cash forecasting, and financial statement preparation, which hinder the effective management of financial resources. Furthermore, adherence to financial management standards was found to be inconsistent, further exacerbating financial challenges. Kandi et al. [70] advocated for local NGOs to engage local fundraisers to reduce their dependency on foreign donors. This aligns with Milelu's [3] findings, which highlighted the importance of internal control measures in promoting donor accountability and strengthening financial reporting systems. According to Milelu, establishing such controls not only ensures the proper use of donor funds but also enhances donor confidence, thereby improving financial sustainability.

In the Lebanese context, Makdissi et al. [71] emphasized that the internal governance of NGOs plays a crucial role in their financial sustainability. While Lebanese NGOs contribute significantly to sustainable development by promoting democratic engagement and good governance, some organizations struggle with internal governance challenges, such as insufficient financial control mechanisms and funding constraints. These internal governance issues can hinder an NGO's ability to manage finances effectively and, consequently, affect its ability to raise and manage funds sustainably.

The aforementioned studies suggest that sound financial management practices, including income diversification, financial control procedures, staff engagement in financial processes, and robust internal governance, are essential for the financial sustainability of NGOs. However, the relationship between financial management practices and fundraising levels remains underexplored in the literature, particularly in the Lebanese NGO sector. Given the increasing financial demands and the critical role NGOs play in Lebanon during times of crisis, examining how financial management practices impact their ability to secure funds is crucial. Thus, the following hypothesis is proposed.

H4 The fundraising levels of Lebanese NGOs are affected by financial management practices.

**Fig. 1:**
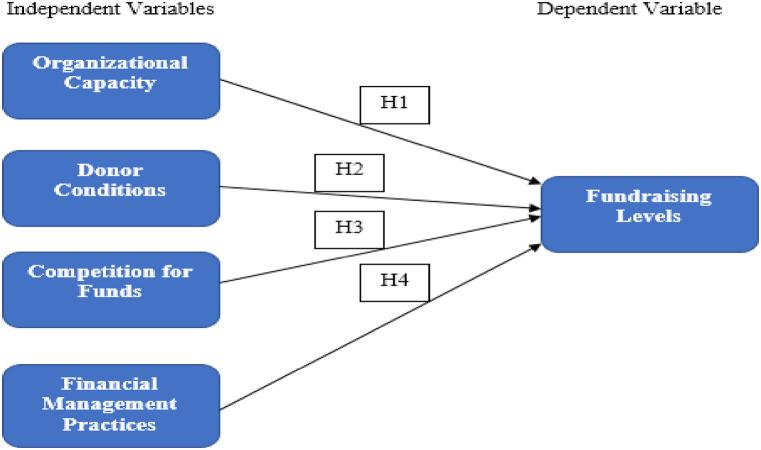
Conceptual framework.

The conceptual model grouping the variables considered in this study and the hypothesized relationship between them is shown in Fig. 1.

## 3 Methodology

### 3.1 Research design

The research design adopted in this study is primarily explanatory, utilizing a positivist and objectivist philosophy to maintain objectivity and impartiality. The research philosophy is firmly rooted in positivism, emphasizing rigorous scientific methods for empirical and quantifiable data collection and analysis. The deductive research approach begins with a theoretical foundation, reviewing existing literature, and formulating testable hypotheses. Moreover, the chosen research approach is a mono-method quantitative design, emphasizing numerical data and statistical analysis for its objectivity, generalizability, efficiency, and clear interpretation.

### 3.2 Research strategy

The survey strategy was employed to capture essential data for research objectives. The structured questionnaire includes three sections where the first includes sociodemographic data, the second covers organizational profiles of NGOs, and the third is dedicated to testing the hypotheses.

The demographic data include the gender, age, educational level, position in the NGO, years of experience, and the extent of responsibility of the respondent in the NGO's fundraising activities.

The second section includes the number of years of the operation of the NGO, the NGO's source of funding, kind of funding for foreign funds, kind of funding for local funds, primary contributors to the NGO's funding, amount of an average typical donation, availability of sufficient sources of funding to pursue the NGO's objective, trend of foreign funding, and reasons for donors to contribute. The questions in this section were multiple-choice questions.

The third section includes Likert scale agreement statements related to the variables of the study. To measure the organizational capacity of NGOs, statements intended to examine whether NGOs have professional fundraising staff, the involvement of their executive directors in fundraising activities, and the availability of necessary assets (such as equipment) for supporting fundraising. Additional aspects include whether the organization hires fundraising consultants, engages in media outreach to potential donors, and exercises cautiousness in receiving funds from donors. Also, statements were used to evaluate the presence of a proven track record, the leadership's ability to influence donors, and the establishment of fundraising strategies. To measure donor's funding conditions, statements investigated the complexity and consistency of donor funding conditions, issues such as how donors require organizations to utilize funds for specific purposes, the educational requirements imposed by foreign donors, donor monitoring and appraisal practices, financial management training, deadlines for fund utilization, and reporting requirements. To measure competition for funds, respondents were asked to provide insights into the competition for fundraising resources. The statements explore whether NGOs have adequate resources to compete with international NGOs and other national NGOs and how competition manifests in terms of beneficiaries and politically affiliated NGOs. It also examines how organizations manage competition for limited resources within the sector. For financial management practices, statements were added to assess the effectiveness of NGOs' financial management systems. It focuses on whether organizational boards authorize budgets annually, the role of finance staff in reviewing financial documents, the implementation of budgeting and monitoring processes, and the regular review of financial systems by donors. Statements also cover the use of cash forecasts for planning and the application of computerized accounting software for financial reporting. Finally, to assess fundraising levels, participants were asked to provide their agreement on whether organizations follow proactive fundraising plans, can implement new strategies, leverage experienced board members for donor relationships, and set realistic missions, visions, goals, and objectives for fundraising efforts. This structured approach offers a comprehensive assessment of the factors influencing NGOs' fundraising capacity, donor conditions, competition for funds, financial management, and overall fundraising success.

### 3.3 Population and sample selection

Cross-sectional data was collected via Google Forms, and the survey was distributed to individuals holding pivotal roles in NGOs, including fundraising managers, program managers, finance managers, and executive directors. These positions were carefully selected due to their direct involvement in organizational fundraising strategies and financial management practices. Therefore, the chosen sample provides unique, highly informed insights into the fundraising dynamics and organizational capacity of NGOs in Lebanon. While the obtained sample size of 124 participants may appear modest relative to the reliable size as per Qualtrics which shows a convenient sample of 373 NGOs out of the population of 11,676, it reflects a targeted selection of key leadership roles that are pivotal to understanding the study's focus. By gathering data from these influential positions, the study ensures that the responses are not only reflective of individual perceptions but also grounded in strategic decision-making that directly influences NGO fundraising capacity. This approach prioritizes the quality and depth of insights over a larger, more generalized sample, and thus allows for stronger conclusions to be drawn regarding the specific organizational and fundraising practices of Lebanese NGOs. These respondents are positioned to provide informed perspectives on the challenges and opportunities related to fundraising within their organizations.

### 3.4 Ethical considerations

Informed consent from participants was obtained. Participants were asked a yes/no question at the beginning of the questionnaire, where selecting "yes" indicated their voluntary and informed consent to participate. Also, the anonymity and confidentiality of participants was ensured. Ethical approval was obtained from the Research Ethics Committee (REC) under the approval code HCR/EC 2023-054 on 20-10-2023.

### 3.5 Data analysis

Data analysis techniques included Cronbach's alpha for internal consistency and regression analysis. Based on the conceptual model and hypotheses, the regression model is articulated as follows:

FundraisingLevels=β0+β1(FinancialManagementPractices)+β2(DonorFundingConditions)+β3(FundraisingCapacity)+β4(CompetitionforFunds)+ϵ

The dependent variable is the Fundraising Levels of NGOs and the independent variables are as follows: Financial Management Practices, Donor Funding Conditions, Organizational Fundraising Capacity, and Competition for Funds.

## 4 Outcome of the research and analysis

### 4.1 Demographic variables

Table 1 summarizes the demographic factors included in this study. There are 48.4 % female and 51.6 % male participants. The distribution of their ages is as follows: between the ages of 21 and 30, 39.5 %, 31 to 40, 29 %, 41 to 50, 22.6 % and over 51, 8.9 %. In addition, 3.2 % of the sample is high school graduates, 4.8 % hold a technical baccalaureate degree, 20.2 % hold a superior technique diploma, 37.9 % are bachelor's degree holders, 25 % are master's degree holders, 7.3 % are Ph.D. holders and 1.6 % are Post Docs. Of the participants, 41.1 % hold the position of fundraising manager/development director, 21.8 % are program managers/coordinators, 30.6 % are financial officers and 6.5 % are currently executive directors/Chief Executive Officers. Regarding the work experience of the participants, 6.5 % have less than 1 year of experience, 28.2 % have between one to five years of work experience, 41.9 % have between six to ten years, 19.4 % have between eleven to fifteen years, and 4 % have over 16 years of work experience. Furthermore, 18.5 % of the participants don't have any responsibilities for their organization's fundraising activities, 29 % are partly responsible and 52.4 % are fully responsible.

**Table 1:** Demographic variables.

Demographic Factor	Category	Percentage (%)
**Gender**	Female	48.4
	Male	51.6
**Age**	21–30	39.5
	31–40	29
	41–50	22.6
	51+	8.9
**Educational Level**	High School Graduate	3.2
	Technical Baccalaureate Degree	4.8
	Superior Technique Diploma	20.2
	Bachelor's Degree	37.9
	Master's Degree	25
	Ph.D. Holder	7.3
	Postdoctoral Degree	1.6
**Position in Organization**	Fundraising Manager/Development Director	41.1
	Program Manager/Coordinator	21.8
	Financial Officer	30.6
	Executive Director (Chief Executive Officer)	6.5
**Work Experience**	Less than 1 year	6.5
	1–5 years	28.2
	6–10 years	41.9
	11–15 years	19.4
	Over 16 years	4
**Responsibility for Fundraising**	No Responsibility	18.5
	Partly Responsible	29
	Fully Responsible	52.4

The demographic breakdown of the sample supports the validity of the sample size (124 participants) by highlighting the strategic selection of participants who hold key roles within their organizations. The sample includes a balanced gender distribution, and the age range is diverse, ensuring a mix of perspectives from different career stages. Educationally, the respondents are well-qualified which ensures that the participants are knowledgeable about the issues being studied. Crucially, the majority of participants occupy influential roles in fundraising and decision-making within their organizations which ensures that the insights gathered come from those directly involved in or responsible for fundraising activities. Additionally, 52.4 % of respondents are fully responsible for their organization's fundraising, 29 % are partly responsible, and only 18.5 % have no responsibilities in this area. This concentration of key decision-makers strengthens the ability of the study to draw meaningful conclusions, despite the smaller sample size.

### 4.2 Overview of the NGOs surveyed

To create a thorough picture of the NGOs surveyed, the questionnaire's second section looked at important organizational factors as shown in Table 2.

**Table 2:** Organizational-related profile questions.

Organizational-Related Profile Questions	Percentage (%)
**Years of Operation of NGOs**	
1–5 years	30.60 %
6–10 years	37.10 %
11–15 years	22.60 %
16–20 years	8.10 %
Over 21 years	1.60 %
**Funding Sources**	
Foreign donors	29.00 %
Local donors	33.10 %
Both foreign and local sources	37.90 %
**Foreign Funding Breakdown**	
Donations	40.30 %
Grants	12.10 %
Gifts	14.50 %
**Local Funding Breakdown**	
Income-generating activities	48.40 %
Member contributions	17.70 %
**Primary Contributors**	
Governmental agencies	15.30 %
Corporate donors	37.10 %
Individual donors	23.40 %
International organizations	21.80 %
Donor agencies & aid programs	2.40 %
**Average Donation Amount**	
Less than $100	15.30 %
$100 - $500	35.50 %
$500 - $1000	30.60 %
$1000 - $5000	8.90 %
$5000 - $10,000	4.80 %
Over $10,000	4.80 %
**Sufficiency of NGO Funding**	
Yes	54.80 %
No	45.20 %
**Foreign Funding Trend**	
Increasing	51.60 %
Decreasing	11.30 %
Fluctuating	2.40 %
Stagnating	1.60 %
**Donors' Motivations**	
Moral obligation	5.60 %
Tax incentives (Tax Exemption)	21.00 %
Social impact	14.50 %
Public image enhancement	19.40 %
Personal connections/experience	7.30 %
Political influence	10.50 %
Corporate Social Responsibility (CSR)	13.70 %
Altruism or compassion	4.00 %
Economic benefits or networking	4.00 %

Regarding the years of operation of NGOs, 30.6 % have been in existence for 1–5 years, 37.1 % for 6–10 years, 22.6 % for 11–15 years, 8.1 % for 16–20 years, and 1.6 % for more than 21 years.

10.13039/100014337Furthermore, 29.0 % of surveyed NGOs rely on foreign donors, 33.1 % on local donors, and 37.9 % secure their funding from both foreign and local sources.

As for NGOs' foreign funding sources, 40.3 % rely on donations, 12.1 % on grants, and 14.5 % on gifts.

Moreover, for NGOs local funding sources, 48.4 % rely on income-generating activities and 17.7 % on member contributions.

Regarding NGOs' primary contributors, 15.3 % receive support from governmental agencies, 37.1 % from corporate donors, 23.4 % from individual donors, 21.8 % from international organizations, and 2.4 % from donor agencies and aid programs.

As for NGOs' average donation amount, 15.3 % reported receiving less than $100, 35.5 % receiving $100 - $500, 30.6 % obtaining $500 - $1,000, 8.9 % securing $1000 - $5,000, 4.8 % receiving $5000 - $10,000, and another 4.8 % reporting donations exceeding $10,000.

Concerning the sufficiency of NGO funding to pursue organizational objectives, 54.8 % affirmed "Yes" while 45.2 % responded with "No".

The distribution of NGOs based on the trend rate of their foreign funding reveals 51.6 % experiencing an increasing trend, 11.3 % facing a decreasing trend, 2.4 % observing fluctuating trends, and 1.6 % reporting stagnating trends.

Regarding donors' reasons for donating, 5.6 % cited moral obligation, 21.0 % were motivated by tax incentives (Tax Exemption), 14.5 % driven by a desire for social impact, 19.4 % seeking public image enhancement, 7.3 % influenced by personal connections or experience, 10.5 % motivated by political influence, 13.7 % considering Corporate Social Responsibility (CSR), 4.0 % expressing altruism or compassion, and another 4.0 % seeking economic benefits or networking.

### 4.3 Reliability test

According to Table 3, the computed Cronbach's Alpha scores for the variables are greater than 0.7, indicating a good level of reliability for the given data.

**Table 3:** Reliability test.

Variable	Items	Cronbach's Alpha
Organizational Capacity	9	0.955
Donor Funding Conditions	8	0.773
Competition for Funds	6	0.839
Financial Management Practices	6	0.757
Fundraising Levels	4	0.541

### 4.4 Descriptive statistics

#### 4.4.1 Organizational capacity

When evaluating the fundraising capacity of NGOs, respondents expressed diverse perspectives on various dimensions. A substantial majority, comprising 72.6 %, acknowledged the presence of professional fundraising staff. Similarly, concerning executive directors' involvement in fundraising, a significant 76.6 % affirmed active participation. Assessing the adequacy of assets for fundraising, 75.0 % acknowledged sufficiency. Notably, the majority (83.1 %) confirmed the engagement of fundraising consultants. Regarding media awareness efforts, 50.8 % strongly agreed, 25.8 % agreed, and 23.4 % disagreed.

#### 4.4.2 Donor's funding conditions

When evaluating the funding conditions imposed by donors for NGOs, respondents expressed diverse perspectives on various dimensions. A substantial 76.6 % agreed that the donor's funding priorities are multifaceted and altering. 83.1 % agreed that donors necessitate funds to be used for the planned objective. 33.1 % agreed that foreign donors require highly educated employees while 39.7 % disagreed, and 27.4 % were neutral. 76.6 % agreed that donors repeatedly visit the NGO to assess the progress of the program(s) they funded. 56 % disagreed that donors give training on financial management practices and 33.9 % agreed, with 9.7 % adopting a neutral stance. 61.4 % disagreed that donors ask that funds be used within the agreed time, while 30.6 % agreed, and 7.3 % remained neutral. 61.4 % disagree that donors ask for the return of unspent balances while 29.8 % agreed, and 8.9 % took a neutral position. Finally, 59 % disagreed that donors demand various reporting necessities for projects, while 20.2 % agreed, and 20.8 % remained neutral. These varied perspectives highlight the complex terrain of views on donor funding conditions within the surveyed NGOs.

#### 4.4.3 Competition for Funds

When evaluating the competition for funds within NGOs, respondents expressed diverse perspectives on various dimensions. 59.7 % agreed that their NGO has adequate resources to compete with international NGOs, 16.9 % disagreed, and 23.4 % were neutral. When asked about their NGO competition for funds emerging from new national NGO entrants, a majority of 56.5 % agreed, while 27.4 % disagreed, and 16.1 % remained neutral. 77.4 % agreed that there is competition for receivers by both the national and the international NGOs executing the same projects in the same area. 70.1 % agreed that their NGO competes for funds from politically linked or affiliated NGOs. 76.6 % agreed that their NGO competes moderately with other NGOs for limited resources. 56.5 % agreed that their NGO has proper resources to compete with other international NGOs in the fundraising campaign, 27.4 % disagreed, and 16.1 % adopted a neutral stance.

#### 4.4.4 NGO's financial management practices

In exploring the landscape of financial management practices within NGOs, respondents provided a diverse array of perspectives. A unanimous 70.2 % agreed that the NGO's board members annually approve the budget. A notable 75.8 % concurred that finance personnel review all financial statements and budget proposals. 76.6 % agreed that the NGO's management implements monthly budgeting and monitoring. 69.3 % agreed that there is a monthly review of financial information by the funders. 54.1 % disagreed that cash forecast is continuously prepared monthly, quarterly, and annually for cash need planning in project execution while 33.9 % agreed and 3.2 % expressed neutrality. Finally, 65.3 % agreed that proper accounting software is used to perform recording entries and financial reporting.

#### 4.4.5 NGOs fundraising levels

In examining the fundraising levels of NGOs, diverse perspectives emerged among respondents. 79.1 % agreed that the NGO practices a proactive fundraising plan. 56.5 % agreed that their NGO has the resources and capacity to implement new fundraising strategies while 26.9 % disagreed, and 16.1 % were neutral. 85.5 % agreed that their NGO has experienced board members with good relationships with donors with 11.3 % expressing disagreement and 2.4 % taking a neutral position. Lastly, 74.9 % agreed that their NGO sets a realistic mission, vision, goals, and objectives while 24.9 % disagreed and 16.9 % were neutral. These nuanced insights illuminate the diverse perceptions of fundraising levels within the surveyed NGOs.

### 4.5 Summary statistics for all variables

Table 4 shows the summary statistics for all variables of this study. The average perceived fundraising capacity of NGOs has a mean of 3.68 and a median of 4.11. The standard deviation of 0.93 and a wide range of responses (1.78–4.67) suggest notable variability. This may reflect disparities in resources, expertise, or access to funding opportunities among the surveyed NGOs. Donor-imposed funding conditions are perceived moderately by the respondents, with a mean of 3.19 and a median of 3.25. The low standard deviation of 0.52 indicates a relatively consistent perception of these conditions across NGOs. The competition among NGOs for funding is perceived to be moderately high, with a mean of 3.54 and a median of 3.58. The variability is indicated by a standard deviation of 0.71. Financial management practices among NGOs are rated positively, with a mean of 3.73, a median of 3.83, and a standard deviation of 0.76. Finally, NGOs’ overall fundraising levels are perceived with a mean of 3.68 and a median of 3.75. The responses show relatively low variability (standard deviation of 0.64), indicating that most NGOs have a fairly consistent perception of their fundraising outcomes.

**Table 4:** Summary statistics for all variables.

Statistics						
		NGOs Fundraising Capacity	Donor's Funding Conditions	Competition for Funds	NGOs Financial Management Practices	NGOs Fundraising Levels
N	Valid	124	124	124	124	124
	Missing	0	0	0	0	0
Mean		3.6787	3.1916	3.5435	3.7283	3.6821
Median		4.1111	3.2500	3.5833	3.8333	3.7500
Std. Deviation		0.92720	0.51836	0.70748	0.75823	0.64205
Minimum		1.78	2.13	2.00	1.83	2.25
Maximum		4.67	4.25	4.33	4.83	4.75

### 4.6 Regression analysis

In this study of NGOs in Lebanon, the regression analysis provides insight into the factors influencing fundraising levels which are shown in Table 5.

**Table 5:** Regression analysis.

Model Summary				
Model	R	R Square	Adjusted R Square	Std. Error of the Estimate
1	.822^a^	0.675	0.665	0.45365

**ANOVA**						
**Model**		**Sum of Squares**	**df**	**Mean Square**	**F**	**Sig.**
**1**	**Regression**	50.975	4	12.744	61.924	.000^b^
	**Residual**	24.490	119	0.206		
	**Total**	75.465	123			

**Coefficients**						
**Model**		**Unstandardized Coefficients**		**Standardized Coefficients**	**t**	**Sig.**
		**B**	**Std. Error**	**Beta**		
**1**	**(Constant)**	0.885	0.280		3.166	0.002
	**NGOs Fundraising** **Capacity**	0.016	0.093	0.019	0.178	0.859
	**Donor Funding** **Conditions**	−0.038	0.111	−0.026	−0.344	0.731
	**Competition for** **Funds**	0.965	0.161	0.953	5.996	0.000
	**NGOs Financial** **Management** **Practices**	−0.151	0.143	−0.145	−1.053	0.294

a Dependent Variable: NGOs Fundraising Levels (n = 124).

In the model, the correlation coefficient (R) is 0.822, indicating a strong positive correlation between the predictors and NGOs' fundraising levels. The R-squared value is 0.675, suggesting that approximately 67.5 % of the variance in NGOs' fundraising levels can be explained by the predictors included in the model. The adjusted R-squared value of 0.665 accounts for the number of predictors, reaffirming the model's robustness. The standard error is 0.45365, which indicates the average difference between observed and predicted values of NGOs' fundraising levels.

The ANOVA results confirm the model's significance. The F value is 61.924 with a p-value less than 0.001, indicating that the predictors collectively contribute significantly to explaining the variance in NGOs' fundraising levels.

The coefficients table provides insights into the individual contributions of each predictor. The constant term is 0.885 (p-value = 0.002), suggesting a baseline positive impact on NGOs' fundraising levels. Fundraising Capacity is 0.016 (p-value = 0.859 > 0.05), indicating a negligible and non-significant positive relationship with fundraising levels. The coefficient for Donor Funding Conditions is −0.038 (p-value = 0.731 > 0.05), suggesting a negligible and non-significant negative relationship. The coefficient for Competition for Funds is 0.965 (p-value <0.001), indicating a strong and significant positive relationship. This implies that greater competition positively influences fundraising levels. The coefficient for Financial Management Practices is −0.151 (p-value = 0.294 > 0.05), indicating a non-significant negative relationship.

Thus, Competition for Funds is the only significant factor influencing the fundraising levels, as indicated by a p-value of less than 0.001. Thus, the regression equation can be as follows:

NGOs’ Fundraising Levels = 0.885 + 0.965 × Competition for Funds

Conversely, the remaining variables do not show significant effects in this context. The results suggest that NGOs should focus on strategies to enhance their competitiveness in the fundraising landscape.

### 4.7 Discussion

The results of the regression analysis can be directly linked to the hypotheses formulated regarding the factors affecting the fundraising levels of NGOs in Lebanon.

Hypothesis 1 proposed that organizational capacity would significantly impact fundraising levels. However, the regression analysis revealed that NGO fundraising capacity was not a significant predictor (Beta = 0.019, p-value = 0.859). This finding suggests that organizational capacity, in terms of resources and skills specifically related to fundraising, may not be the primary driver of fundraising success in the current Lebanese context. It highlights a potential disconnect between perceived capacity and actual effectiveness in raising funds, indicating that NGOs might need to rethink their approaches to capacity building or focus on other areas that enhance their appeal to donors.

The second hypothesis posited that donor funding conditions would significantly influence fundraising levels. The analysis indicated that donor funding conditions had a negative but non-significant impact (Beta = −0.026, p = 0.731). This finding points to the challenging environment NGOs operate, where external funding conditions may not significantly affect their overall fundraising efforts. Factors such as donor fatigue or shifting priorities in a crisis-laden context could mean that organizations must adapt to a landscape where traditional funding mechanisms are under pressure. Consequently, NGOs may need to explore diverse funding strategies, such as crowdfunding or private-sector partnerships, to mitigate the effects of unfavorable funding conditions.

The third hypothesis received strong support from the analysis, with competition for funds emerging as a significant positive predictor (Beta = 0.953, p < 0.001). This suggests that the level of competition among NGOs significantly drives their fundraising levels. In Lebanon, where many NGOs vie for limited donor resources, those that can effectively communicate their value propositions and impact are more likely to succeed in attracting funds. This finding emphasizes the necessity for NGOs to engage in competitive strategies that can differentiate them in a crowded marketplace, highlighting the importance of effective marketing and outreach in the fundraising process. This result is consistent with existing literature that highlights the challenges faced by NGOs in developing regions. Batti [9] examined local NGOs in Africa and noted that their reliance on foreign donors, combined with stiff competition, often results in insufficient resources to fully implement projects. This observation is pertinent to Lebanon, where NGOs may similarly struggle to achieve financial stability and scale their operations due to fierce competition for limited donor funds. Also, Aden [66] explored the experiences of local NGOs in Somalia, particularly those involved in nutrition projects. He found that intense competition for funding contributed to shortages that led to corruption, bribery, and a lack of coordination among organizations. This disruption not only undermined service delivery but also threatened the long-term sustainability of the NGOs. The findings in Lebanon resonate with Aden's observations, indicating that while the number of NGOs may be substantial, the competition for funds can compromise their effectiveness and operational viability. Mymunah [67] further contributed to this discourse by arguing that competition necessitates the development of robust internal strategies for NGOs. This recommendation aligns with the significant influence of competition for funds found in our analysis, suggesting that Lebanese NGOs must prioritize innovation and develop sustainable revenue models to mitigate the adverse effects of competition.

Finally, the analysis showed that an NGO's financial management practices did not significantly influence fundraising levels (Beta = −0.145, p = 0.294). In the Lebanese context, where financial instability is prevalent, NGOs may need to focus more on strategic financial practices that not only ensure sustainability but also appeal to donors looking for transparency and accountability. The lack of significance here suggests that merely having financial management practices in place is insufficient; NGOs must ensure these practices are effectively implemented and communicated to stakeholders.

Thus, the findings reveal that competition for funds is the only significant factor influencing the fundraising levels of NGOs in Lebanon. The other hypotheses, while relevant, did not find support in this analysis, highlighting the complexity of the fundraising environment. This underscores the necessity for NGOs to focus on competitive strategies and innovative fundraising approaches while potentially reassessing their internal capacities and financial management practices to enhance their overall effectiveness in securing funding. This nuanced understanding of the interplay between these variables can guide NGOs in Lebanon as they navigate a challenging funding landscape.

## 5 Conclusion

The results suggest that Lebanon's context presents unique challenges and dynamics that may explain why certain factors—such as organizational capacity, donor funding conditions, and financial management practices—were found to be insignificant in influencing NGOs' fundraising levels.

Lebanon has been experiencing significant economic turmoil, characterized by hyperinflation, currency devaluation, and a general lack of financial resources. This instability can overshadow the influence of organizational capacity or sound financial management practices, as NGOs may primarily focus on survival rather than long-term planning or capacity building. In contrast, the contexts studied in previous studies discussed in the literature review might have had relatively stable environments, allowing for a greater emphasis on these factors.

Further, Lebanon's NGOs may be heavily reliant on international donors due to a lack of local funding sources. This reliance may diminish the significance of internal factors, such as organizational capacity or financial management practices, as NGOs scramble to adapt to changing donor priorities and competition for limited funds. In contrast, other regions studied may have had more diverse funding sources, allowing for a stronger correlation with organizational capacity and management practices.

In addition, Lebanon's complex political landscape can significantly impact NGOs' operations. Factors such as political patronage, sectarianism, and regulatory challenges might influence fundraising dynamics in ways that were not captured in the studies from Africa or Somalia. For example, political connections may determine funding access more than an NGO's organizational capacity or fundraising strategies, leading to a diminished impact of those factors on overall fundraising levels.

Also, cultural factors may play a role in shaping fundraising dynamics. The expectations of donors, community engagement practices, and perceptions of NGOs can vary significantly by region. In Lebanon, community trust and donor motivations might be influenced more by external factors, such as competition for funds, rather than the internal capabilities of NGOs. Previous studies may not have accounted for these cultural nuances, which could explain the differences in findings.

Given the urgent humanitarian needs in Lebanon, especially amidst the ongoing economic crisis and political turmoil, NGOs may prioritize immediate service delivery over developing long-term strategies or building organizational capacity. This focus on short-term survival can result in lower significance for factors like financial management practices and fundraising capacity compared to the immediate pressures of competition for funds.

In conclusion, the unique socio-economic, political, and cultural context of Lebanon likely contributes to the insignificance of organizational capacity, donor funding conditions, and financial management practices in influencing NGOs' fundraising levels. While previous studies provide valuable insights, the particularities of the Lebanese landscape necessitate a nuanced understanding of how NGOs navigate their operational challenges. This highlights the importance of further research to explore how local contexts influence NGO dynamics and fundraising effectiveness.

## 6 Recommendations

To enhance fundraising effectiveness and sustainability among NGOs in Lebanon, several strategic recommendations are essential, each accompanied by practical implementation strategies supported by real-world examples.

First, NGOs should prioritize developing competitive fundraising strategies that adapt to the evolving funding landscape. This includes diversifying funding sources by exploring social enterprises—establishing businesses whose profits are reinvested into the organization's mission. For instance, Kiva is a non-profit organization that connects lenders to entrepreneurs and students in over 80 countries through an online platform. Founded in 2005, Kiva has created a platform for micro-lending, allowing individuals to contribute to various projects globally. Kiva allows individuals to lend money to small businesses and community projects via microloans, often as little as $25. Lebanese NGOs can develop similar local micro-lending platforms tailored to their communities' needs, generating revenue while addressing social issues.

Additionally, enhancing organizational capacity is crucial for improving fundraising skills and project management. NGOs can invest in training and development programs for staff by collaborating with universities or professional organizations to design workshops. For example, Lebanon & Beyond provides capacity-building programs that equip NGOs with essential skills in project management and fundraising. Implementing mentorship programs, where experienced fundraising professionals guide junior staff, can further strengthen organizational capacity. Furthermore, fostering partnerships and collaborations with other NGOs, local businesses, and community organizations can amplify fundraising efforts. By partnering with local businesses for sponsorship or co-hosting community events such as fundraising fairs or charity runs, NGOs can leverage resources and reach wider audiences. A successful case study is the Lebanese Red Cross, which frequently collaborates with various stakeholders for blood donation drives and fundraising events, maximizing outreach and impact.

Moreover, NGOs should adopt a donor-centered approach that prioritizes communication and engagement with donors. Implementing donor management software can help track contributions and personalize communication. UNICEF, for example, uses a donor engagement strategy that includes personalized updates, impact stories, and recognition initiatives. Lebanese NGOs can adopt similar practices by regularly updating donors about project outcomes and expressing gratitude through personalized messages or events. Advocacy for supportive policies is another vital recommendation; active engagement in advocacy efforts can influence policies that benefit the NGO sector. By collaborating with networks like Lebanon's National Council for Non-Governmental Organizations, NGOs can lobby for favorable legislation. A concrete example is the successful advocacy by NGOs in South Africa, which resulted in tax deductions for charitable donations. Lebanese NGOs can promote similar legislation to incentivize local donations.

In addition, embracing technology is essential for enhancing fundraising capabilities. NGOs can utilize data analytics to identify potential donor segments, similar to how platforms like GoFundMe effectively target campaigns. Investing in donor management systems can streamline communication and improve donor engagement. Conducting workshops to train staff on using Customer Relationship Management (CRM) tools such as Salesforce or HubSpot can also be beneficial. Furthermore, conducting regular needs assessments will help NGOs align their fundraising strategies with community priorities. This can be implemented through surveys or focus groups to gather input from beneficiaries and stakeholders. A practical example is the World Food Program (WFP), which conducts assessments to understand community needs and tailor its programs accordingly. Lebanese NGOs can engage their communities in discussions to identify pressing issues and shape their fundraising campaigns around these insights.

Establishing strong governance and accountability mechanisms is crucial for building trust with donors. NGOs should adopt best practices by providing regular financial reports and impact assessments. A successful model can be seen in Oxfam, which publishes detailed annual reports outlining financial statements and project impacts. Lebanese NGOs should establish similar transparency measures by sharing reports on their websites and engaging donors in discussions about financial accountability. Finally, leveraging evidence-based advocacy by using data to support fundraising efforts is essential. NGOs can conduct impact assessments and utilize the findings to advocate for their initiatives, similar to the Gates Foundation, which uses data-driven insights to influence global health funding. Lebanese NGOs can publish research reports showcasing the effectiveness of their programs, thereby attracting interest from both local and international donors.

By implementing these recommendations, Lebanese NGOs can strengthen their fundraising capabilities, enhance organizational resilience, and ultimately improve their capacity to deliver essential services to the communities they serve.

## 7 Limitations and future research

This study acknowledges several limitations that may influence the generalizability and depth of its findings. First, while the sample comprised key decision-makers in NGOs across Lebanon, it may not sufficiently represent the broader landscape of NGOs operating in the country. The diversity in organizational types, sizes, and missions is vast, and a larger, more varied sample could yield a more nuanced understanding of fundraising practices. Future research should aim to incorporate a wider range of NGOs, including grassroots organizations and those operating in different sectors, to capture a comprehensive picture of fundraising dynamics across the sector.

Secondly, the analysis was constrained by the limited scope of variables examined. While factors such as donor funding conditions, financial management practices, fundraising capacity, and competition for funds were included, other potentially influential variables—such as the role of digital fundraising strategies, volunteer engagement, and community support—were not considered. Future studies could benefit from a broader array of variables, allowing for a more intricate exploration of the determinants that influence fundraising success for NGOs.

Furthermore, the unique socio-economic context of Lebanon, characterized by ongoing economic crises and political instability, may significantly affect fundraising levels. The current climate creates a volatile environment for NGOs, and understanding how these external pressures shape fundraising strategies would provide valuable insights. Future research should consider longitudinal studies to examine how fundraising practices adapt in response to shifting political and economic landscapes.

Lastly, this research predominantly employed quantitative methods, which, while effective for assessing statistical relationships, may limit the depth of qualitative insights into fundraising dynamics. A mixed-methods approach in future research could enrich the understanding of fundraising practices by integrating qualitative data, such as interviews or focus groups, which can provide deeper contextual insights and personal narratives. This comprehensive perspective would offer a more robust exploration of the factors influencing fundraising outcomes and could uncover nuanced insights that quantitative methods alone might overlook.

In summary, addressing these limitations through expanded sampling, diverse variable consideration, and methodological diversification will enhance the understanding of NGO fundraising dynamics, leading to more actionable recommendations for practitioners in the field.

## 8 Contributions

This study significantly contributes to the existing literature on NGO fundraising by specifically addressing the unique context of Lebanon, a region often overlooked in prior research. By focusing on Lebanese NGOs, this study fills a critical gap in understanding how local factors, such as economic crises and political instability, influence fundraising practices. The findings challenge existing theories by revealing that while competition for funds is a significant determinant, other commonly cited factors—such as financial management practices and donor funding conditions—may not hold the same weight in this context.

Moreover, the study highlights the necessity for a localized understanding of fundraising dynamics, suggesting that strategies effective in other regions may not be directly applicable to Lebanon's distinctive environment. By employing a rigorous quantitative analysis, the research offers valuable insights for practitioners and policymakers, providing a foundation for tailored fundraising strategies that reflect the realities of the Lebanese nonprofit sector.

In terms of originality, this research integrates a mix of established theories with a focus on Lebanon's socio-economic context, advancing the discourse on NGO fundraising in developing regions. By identifying the interplay between competition and fundraising success, the study lays the groundwork for future inquiries into the strategic adaptations necessary for NGOs to thrive in challenging environments. Finally, this study underscores the importance of context-specific research in the global nonprofit landscape, paving the way for future investigations that consider the unique challenges faced by NGOs in similar settings.

During the preparation of this work, the authors used Grammarly to improve language and readability. After using this tool, the authors reviewed and edited the content as needed and took full responsibility for the content of the publication.

## CRediT authorship contribution statement

**Nada Jabbour Al Maalouf:** Writing – original draft, Formal analysis, Writing – review & editing. **Chadia Sawaya:** Methodology, Writing – original draft. **Jean Elia:** Writing – original draft, Conceptualization, Methodology.

## Declaration of competing interest

The authors declare that there are no financial/personal interests or beliefs that could affect their objectivity.

The authors declare that there are no funding or research grants received in the course of study, research, or assembly of the manuscript.

The authors declare that the manuscript was not previously published.

The authors declare that the manuscript is not under consideration for publication elsewhere.

The article's publication is approved by all authors.
